# Moderate Dietary Supplementation with Omega-3 Fatty Acids Does Not Impact Plasma Von Willebrand Factor Profile in Mildly Hypertensive Subjects

**DOI:** 10.1155/2015/394871

**Published:** 2015-08-02

**Authors:** Corinna S. Bürgin-Maunder, Peter R. Brooks, Deborah Hitchen-Holmes, Fraser D. Russell

**Affiliations:** ^1^Inflammation and Healing Research Cluster, University of the Sunshine Coast, Maroochydore, 4556 QLD, Australia; ^2^School of Health and Sport Sciences, University of the Sunshine Coast, Maroochydore, 4556 QLD, Australia; ^3^School of Science and Engineering, University of the Sunshine Coast, Maroochydore, 4556 QLD, Australia; ^4^School of Nursing and Midwifery, University of the Sunshine Coast, Maroochydore, 4556 QLD, Australia

## Abstract

Long chain omega-3 polyunsaturated fatty acids (LC n-3 PUFAs) have blood pressure lowering and antithrombotic effects, which may benefit hypertensive patients. Increased plasma concentration of von Willebrand factor (vWF), a procoagulant glycoprotein, has been identified in patients with severe hypertension, with some, but not all studies showing an increase with mild hypertension. In this study, we determined the plasma concentration, multimer distribution, and collagen binding activity of vWF in subjects with mild hypertension and determined whether these parameters might improve after dietary supplementation with moderate amounts of LC n-3 PUFAs. Hypertensive and normotensive subjects were randomized to 12-week treatment with LC n-3 PUFAs (2.52 g/day) or placebo (canola oil). Home blood pressure measurements were recorded daily, and blood samples were collected every 3 weeks. LC n-3 PUFAs increased the n-3 index to cardioprotective levels (>8%). Plasma concentration, multimer distribution, and collagen binding activity of vWF were not reduced by LC n-3 PUFA treatment. We conclude that, at the concentration and duration used in this study, benefits of LC n-3 PUFAs in subjects with mild hypertension are not associated with a direct effect on vWF concentration or function. This trial is registered with the Australian New Zealand Clinical Trials Registry ACTRN12610000713099.

## 1. Introduction

Hypertensive disease is a risk factor for the development of thrombotic cardiovascular conditions and is a leading cause of death globally [1, 2]. For every 20 mmHg systolic or 10 mmHg diastolic increase in blood pressure, there is reported doubling of mortality from both ischemic heart disease and stroke [2]. The large procoagulant glycoprotein von Willebrand factor (vWF) is produced in vascular endothelial cells and is stored in specific storage granules called Weibel-Palade (WP) bodies [3]. Shear stress and shear-stress induced transcription factor (Krüppel-like factor 2) are associated with a small but significant reduction in number of WP bodies [4], consistent with their degranulation, and increase in basal and adrenaline-stimulated vWF secretion [5]. Hypertensive disease causes an increase in intramural pressure which leads to mechanical shear stress on the endothelium [6]. Accordingly, increased plasma levels of vWF have been reported for patients with stage 2 hypertension (≥160/≥100 mmHg) [7]. The capacity of mild (stage 1) hypertension (140–159/90–99 mmHg) to modulate vWF levels is inconclusive, with an increase in vWF concentration reported by some [8], but not by others [9].

vWF is a multimeric protein, where ultra-large multimers >10,000 kDa have greater thrombogenic potential compared to low molecular weight vWF multimers [10]. The greater thrombogenic potential of the ultra-large multimers is attributed to a large number of binding sites for interactions with platelets, extracellular matrix components and endothelial cells. Since hypertension is linked to an increase in thrombogenic potential, we hypothesized that it might also be associated with a shift in distribution towards larger, more thrombogenic vWF multimers.

Long chain omega-3 polyunsaturated fatty acids (LC n-3 PUFAs) have greater blood pressure-lowering effects in untreated hypertensive patients than in normotensive subjects [11]. We recently showed that LC n-3 PUFAs also modulate the release of vWF from human cultured umbilical vein endothelial cells in a cellular model of vascular inflammation [12]. It is not known whether dietary supplementation of mildly hypertensive subjects with LC n-3 PUFAs will also modulate vWF expression and function. To examine this question, we assessed the effect of 12 weeks of dietary supplementation of mildly hypertensive and normotensive subjects with LC n-3 PUFA (2.52 g/day) on vWF concentration, vWF multimer distribution, and vWF function. The findings showed that whilst the supplement produced cardioprotective levels of LC n-3 PUFAs in membrane phospholipids (n-3 index > 8%), the intervention was not sufficient to modulate plasma vWF concentration, multimer distribution, or collagen binding. It is possible that higher doses of LC n-3 PUFAs are required to modulate vWF in this population or that patients with more advanced hypertensive disease are required to see an effect.

## 2. Materials and Methods

### 2.1. Study Design

The study was conducted as a double-blinded, placebo controlled trial. Age matched normotensive subjects (blood pressure <120/80 mmHg, *n* = 27) and subjects with stage I hypertension (SBP 132–154 mmHg and/or DBP at 63–103 mmHg, *n* = 15) were recruited from March 2009 to September 2010. Each subject participated in the study for a 15-week period, with commencement of treatment from April 2009 to October 2010. Although registration of the trial occurred after commencement of the study (Australian New Zealand Clinical Trial Registry, ACTRN12610000713099), no changes were made to the study design or protocol in the time prior to and time after registration. Subjects were excluded if they were receiving antihypertensive treatment prior to or during the study. All subjects were assigned to placebo capsules (4 × 1 g; Blackmores canola oil) for the first 3 weeks and then randomised to placebo or LC n-3 PUFA supplementation (4 × 1 g; Blackmores Omega Heart, each containing 420 mg EPA and 210 mg DHA; 2.52 g/day) for a 12-week period. This study was conducted in accordance with the Declaration of Helsinki (1964). The protocol was approved by the University of the Sunshine Coast Human Research Ethics Committee (Ethics Approval Number A/08/167). All subjects provided informed, written consent for their participation.

### 2.2. Blood Processing

Blood samples were collected from subjects at 3-week intervals. EDTA and serum tubes were centrifuged (1500 ×g, 15 min, 15°C), and plasma or serum were stored at −80°C until further analysis. Erythrocytes from EDTA tubes were washed with an EDTA solution (1 mL; 0.15 M NaCl, 10 *μ*M EDTA disodium salt, pH 7.4 with HCl), covered with nitrogen gas, and centrifuged at 4°C for 10 minutes (1500 ×g). The process was repeated twice. Aliquots of washed erythrocytes were covered with nitrogen gas and stored at −80°C until further analysis.

### 2.3. Omega-3 Index

To extract phospholipids from the subjects' erythrocytes, 600 *μ*L of methanol containing butylhydroxytoluene (BHT, 20 mg/100 mL) was added to 300 *μ*L of erythrocytes and cells were homogenised using glass rods for 1 min. Homogenates were covered with nitrogen gas and stored on ice for 30 min before adding 600 *μ*L of chloroform. Cells were homogenised again for 1 min, stored on ice for 30 min, and then centrifuged (3000 ×g, 4°C, 5 min). The supernatant was withdrawn, covered with nitrogen gas, and stored on ice. The process was repeated twice using 300 *μ*L methanol with BHT and chloroform, with 10 min storage on ice. To complete the extraction, 800 *μ*L of chloroform and 460 *μ*L of 0.05 M KCl were added to 1000 *μ*L of the pooled lipid solution, mixed by vortex, and centrifuged (3000 ×g, 4°C, 10 min). The supernatant was discarded and the lipid fraction dried under nitrogen gas. To hydrolyse the extracted lipids, 500 *μ*L of 9 M HCl : H_2_O : acetonitrile (1 : 1 : 18) solution containing BHT (25 mg/50 mL) was added, and samples were covered with nitrogen gas and incubated overnight (65°C). Hydrolysed samples were dried under nitrogen gas and freeze-dried for 15 min before adding 250 *μ*L of hexane and 10 *μ*L of derivatising agent (1-tert-butyldimethylsilylimidazole). Samples were covered with nitrogen gas, incubated at 37°C for 2 h, and analysed using a Varian 3900 gas chromatograph (GC) coupled to a Varian Saturn 2100T mass spectrometer (MS). Samples were run on a Zebron ZB-5HT column (30 m × 0.25 mm ID × 0.25 mm film) with 70 eV ionization to determine cellular uptake of EPA and DHA. The n-3 index for each sample was calculated as a proportion of the combined integrated peak areas of DHA and EPA over the total peak area of fatty acids within the sample.

### 2.4. Von Willebrand Factor Plasma Concentration

Plasma vWF concentration was analysed using the IMUBIND vWF ELISA kit (Sekisui Diagnostics LLC, CT, USA).

### 2.5. Von Willebrand Factor Multimer Analysis

Plasma samples were defrosted at 37°C for 5 min and mixed at a 1 : 1 ratio with sample buffer (70 mM Tris base, 4 mM EDTA disodium salt, 9 M urea, 2.4% wt/vol sodium dodecyl sulphate (SDS) and bromophenol blue; pH 6.8). Samples were loaded onto 1% wt/vol horizontal agarose gels (SeaKem HGT-P) made up in gel buffer (100 mM Tris base, pH 8.8, 100 mM glycine and 0.4% wt/vol SDS) and run at 15 mA and 4°C using precooled HE33 mini horizontal agarose electrophoresis units (Hoefer, MA, USA) filled with running buffer cooled to 4°C (50 mM Tris base, 384 mM glycine and 0.1% wt/vol SDS, pH not adjusted; ~8.6) until the dye front reached the bottom of the gels (usually 6 h). Immobilon-P PVDF 0.45 *μ*m membranes (Millipore Australia Pty. Ltd) were equilibrated in methanol (1 min) and then transfer buffer (25 mM Tris base, 192 mM glycine, 20% methanol, and 0.1% SDS, pH not adjusted; ~8.8; 15 min). Gels were equilibrated in transfer buffer for 15 min. A Hoefer TE 62 transfer electrophoresis unit was set up in an ice bath and filled with transfer buffer cooled to 4°C. The transfer was run at 200 Watts for 2 h, with constant stirring. Protein bands were detected using a Vectastain Elite ABC system staining kit, rabbit IgG (Vector Laboratories Inc., CA, USA) and the peroxidase substrate 3,3′-Diaminobenzidine tetrahydrochloride (DAB), with metal enhancer (SIGMA* FAST*). Sixteen bands were resolved, with unresolved very high molecular weight vWF (> band 16) also visible. Band intensity was quantified using ImageJ software (NIH, Bethesda, USA) after subtraction of background from an adjacent area of the membrane for each lane.

### 2.6. Collagen Binding Assay

Binding of vWF to collagen type I was assessed using the TECHNOZYM vWF:CBA ELISA Collagen Type I kit (Technoclone GmbH, Vienna, Austria). 

### 2.7. Blood Pressure Measurements

Home blood pressure measurements were chosen for this study as they provide more accurate cardiovascular risk prediction than office-based clinical blood pressure measurements [13]. The threshold SBP and DBP defining stage 1 hypertension (135–137/86–89 mmHg) are lower for home compared to office blood pressure measurements (140/90) [14]. Subjects were provided with training in the correct method for acquisition of blood pressure data using a Medel Check automated blood pressure device. Four replicate measurements were collected each morning with exclusion of the first measurement. Subjects voided the bladder and abstained from caffeine prior to measurements. SBP and DBP were determined at baseline (7 consecutive days prior to randomization) and at week 12 (final 7 consecutive days of treatment).

### 2.8. Serum Lipid Analysis

Serum concentration of total cholesterol (TC), triglycerides (TG), and high density lipoprotein cholesterol (HDL-C) were determined using enzymatic Infinity kits (Thermo Fisher Scientific Australia Pty Ltd, Vic, Australia). Serum low density lipoprotein cholesterol (LDL-C) was calculated using the Friedewald Formula [15].

### 2.9. Statistical Analysis

Data were analysed using one-way ANOVA, with Tukey's post hoc analysis (IBM SPSS Statistics software, Version 22) or with *t*-tests (Microsoft Excel, Version 14.4.1).

## 3. Results 

### 3.1. Subject Demographics

Subjects recruited to this study were matched for age and gender and, where possible, use of medication and smoking status (Table 1). The mean baseline BMI of subjects in the hypertensive LC n-3 PUFAs group (*n* = 6) was significantly higher when compared to subjects in the normotensive LC n-3 PUFAs treatment group (Table 1, one-way ANOVA, *P* < 0.05, *n* = 9). As expected, mean baseline SBP and mean baseline DBP were significantly higher in hypertensive subjects when compared to normotensive subjects (Table 1, one-way ANOVA, *P* < 0.001). Treatment of hypertensive subjects with the n-3 PUFAs had no significant effect on SBP or DBP (Table 1). There was no difference in serum lipid levels between the groups at baseline and after 12-week treatment with LC n-3 PUFAs (Table 1).

### 3.2. Omega-3 Index Calculations

**Figure 1:**
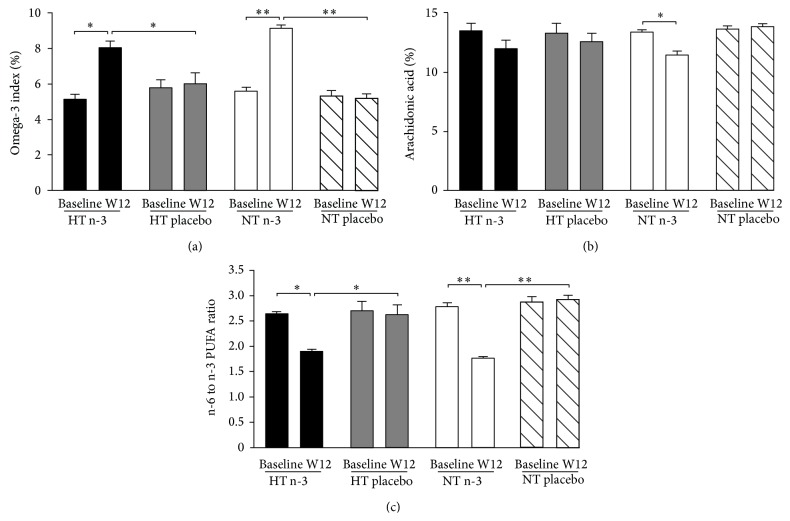
Measurement of the incorporation of long chain polyunsaturated fatty acids (LC PUFA) into erythrocyte membrane phospholipids at baseline and after 12 weeks of treatment with 2.52 g/day n-3 PUFAs. Parameters measured were the omega-3 index (a), the percent arachidonic acid (b), and the n-6 to n-3 PUFA ratio (c) (one-way ANOVA, ^*^: *P* < 0.05; ^**^: *P* < 0.001). Data are expressed as mean ± SEM. W12, Week 12; HT, hypertensive; NT, normotensive; n-3, omega-3 LC PUFA.

The n-3 index was similar for hypertensive and normotensive groups at baseline. As expected, the n-3 index was significantly higher after 12-week n-3 PUFA supplementation when compared to baseline (Figure 1(a), one-way ANOVA, *P* < 0.05). Subjects allocated to the placebo capsules showed no enrichment of their erythrocyte membranes with n-3 PUFAs (Figure 1(a)). Treatment with LC n-3 PUFAs significantly reduced the proportion of arachidonic acid (AA) to all other FAs detected in the erythrocyte membrane phospholipids (% arachidonic acid) in normotensive subjects (Figure 1(b), one-way ANOVA, *P* < 0.05, *n* = 13). However, this was not observed in mildly hypertensive subjects (Figure 1(b)). Additionally, 12-week treatment with LC n-3 PUFAs significantly lowered the n-6 to n-3 PUFA ratio (Figure 1(c), one-way ANOVA, *P* < 0.05).

### 3.3. vWF Plasma Concentration

**Figure 2:**
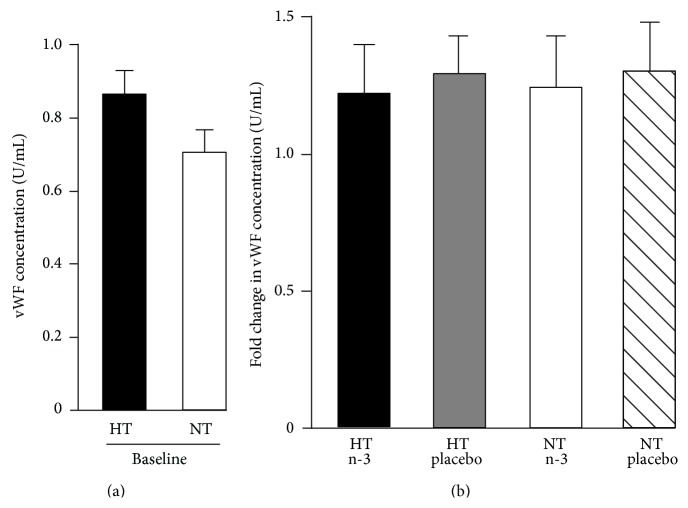
Plasma von Willebrand factor (vWF) concentration at baseline and after 12 weeks of treatment with 2.52 g/day long chain omega-3 polyunsaturated fatty acids (LC n-3 PUFAs). Initial vWF concentration (international units/mL) was not different for hypertensive and normotensive subjects (a). The 12-week supplementation protocol with LC n-3 PUFAs had no effect on plasma concentration of vWF in normotensive and hypertensive subjects (b). Data are expressed as mean ± SEM. HT, hypertensive; NT, normotensive; n-3, LC n-3 PUFAs.

There was no significant difference in plasma vWF concentration between hypertensive and normotensive subjects at baseline (Figure 2(a)). 12-week supplementation with LC n-3 PUFAs or placebo had no effect on vWF plasma concentration in normotensive or hypertensive subjects (Figure 2(b)).

### 3.4. vWF Multimer Analysis

**Figure 3:**
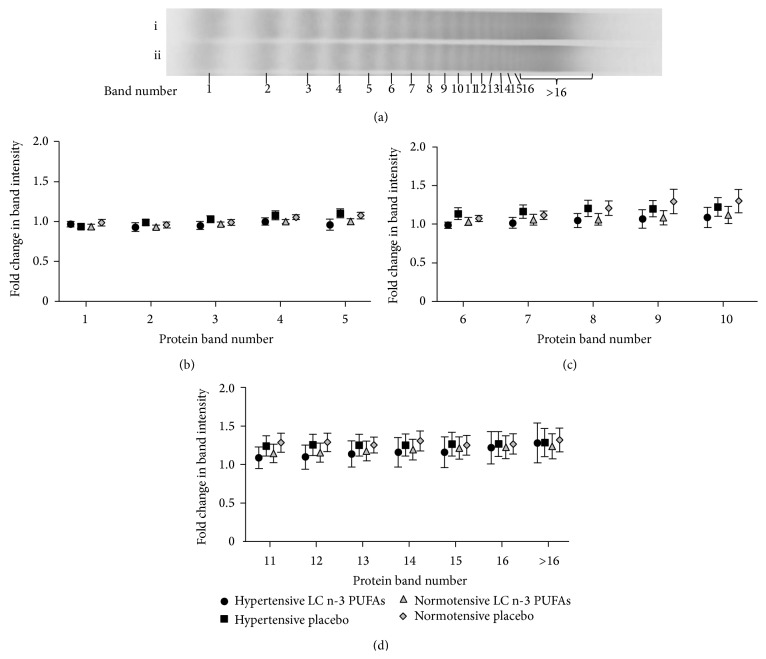
Fold change in von Willebrand factor (vWF) multimer band intensity after 12 weeks of treatment with LC n-3 PUFAs. Representative blots show protein bands at baseline (a)i and after 12 weeks (a)ii. Treatment with placebo or LC n-3 PUFAs had no effect on band intensity of low molecular weight (LMW) vWF (b, bands 1–5), intermediate molecular weight (IMW) vWF (c, bands 6–10), or high molecular weight (HMW) vWF (d, bands > 10) in hypertensive or normotensive subjects. LC n-3 PUFAs had no effect on the fold change of band intensity. Data are expressed as mean ± SEM.

The change in pattern of subjects' vWF multimers throughout the 12-week study period was analysed. There was no significant difference in intensity of LMW (Figure 3(b), bands 1–5), IMW (Figure 3(c), bands 6–10), or HMW (Figure 3(d), bands >10) vWF bands after 12 weeks when compared to baseline within each treatment group (presented as fold change, Figure 3). Exposure to LC n-3 PUFAs or placebo for 12 weeks had no effect on the fold change of LMW (Figure 3(b)), IMW (Figure 3(c)), or HMW vWF band intensity (Figure 3(d)).

### 3.5. Collagen Binding Assay

**Figure 4:**
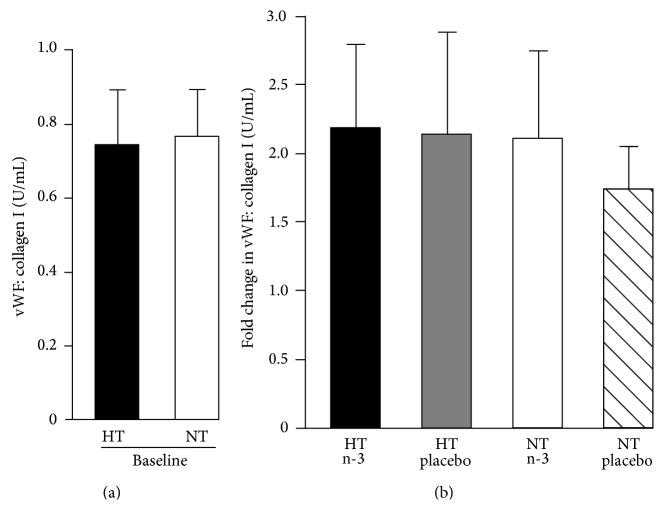
Effect of long chain omega-3 polyunsaturated fatty acid (LC n-3 PUFA) supplementation on von Willebrand factor (vWF) binding to collagen type I. There was no difference in vWF binding (international units/mL) to collagen between hypertensive and normotensive subjects at baseline (a). Twelve-week treatment of subjects with LC n-3 PUFAs or placebo had no effect on vWF binding to collagen (b). Data are expressed as mean ± SEM. HT, hypertensive; NT, normotensive; n-3, LC n-3 PUFA.

Physiological function of vWF was assessed using a collagen type I binding assay. No difference in vWF function was observed between hypertensive and normotensive subjects at baseline (Figure 4(a)). 12-week treatment of subjects with LC n-3 PUFAs or placebo had no effect on vWF binding to collagen (Figure 4(b)).

## 4. Discussion

Treatment of normotensive and hypertensive subjects with LC n-3 PUFAs resulted in a significant increase in incorporation of EPA and DHA into erythrocyte membranes. Epidemiological studies conducted in the USA and Japan showed that an n-3 index < 4% is associated with a 10-fold increase in risk of sudden cardiac death compared to an n-3 index of >8% [16]. In the current study, 12-week treatment increased the n-3 index to cardioprotective levels (>8%). The n-6 : n-3 PUFA ratio was significantly reduced after 12-week treatment with LC n-3 PUFAs. The modern western diet contains high amounts of n-6 PUFAs resulting in a high n-6 to n-3 ratio (~15 : 1), which may promote the development of cardiovascular disease [17]. Increased consumption of n-3 PUFAs lowers the n-6 to n-3 ratio and a dietary ratio of 4 : 1 can reduce mortality from cardiovascular disease [17].

The effect of mild hypertension on vWF levels is controversial. Whilst one study reported an increase of vWF in patients with mild hypertension [8], another did not [9]. We report here that subjects with mild hypertension did not present with higher plasma vWF levels.

Endothelial cells contain granules (WP bodies) that store large amounts of ultra-large vWF multimers. Increased fluid shear stress can induce WP body degranulation [4, 5] and it is therefore possible that endothelial cells exposed to high shear stress during hypertension release this vWF into the vascular lumen [18]. The ultra-large vWF multimers are prone to cleavage, which could result in changes to the distribution of multimeric vWF. In the current study we observed no significant difference in intensity of LMW, IMW, or HMW vWF bands after 12-week LC n-3 PUFA treatment when compared to baseline. Although change in vWF concentration and change in vWF multimer profile were similar across treatment groups and intragroup variability was very small, it might be of interest in future studies to investigate larger cohorts of subjects or a population of subjects with more severe hypertension. It is noteworthy however that severe hypertension is typically managed with blood-pressure lowering medications and that this affects vWF profile [7, 19].

HMW vWF has greater thrombogenic potential than IMW or LMW vWF. vWF function can be assessed using a collagen type I binding assay. No difference in vWF function was observed between hypertensive and normotensive subjects at baseline. 12-week treatment of subjects with LC n-3 PUFAs or placebo had no effect on vWF binding to collagen. Whilst we excluded subjects who were taking antihypertensive medications and subjects who had a high dietary intake of LC n-3 PUFAs, we made no further exclusions based on other medications that subjects might be taking. It is possible that some of these might have impacted on vWF expression or function.

Blood pressure lowering effects of n-3 PUFAs have been reported in some, but not all studies. Responsiveness of blood pressure to n-3 PUFAs is likely influenced by disease state (n-3 PUFAs have greater blood pressure-lowering effects in untreated hypertensive patients than in normotensive subjects; [11]), dose (Higher doses of n-3 PUFAs are required for responses such as blood pressure reduction, whereas lower doses are sufficient for responses such as improved myocardial oxygen efficiency; [20]), and formulation (n-3 PUFAs, but not their ethyl ester derivatives, activate BK channels to reduce blood pressure; [21]). In this study, a nonsignificant trend for reduced SBP (4.5 mmHg) was detected in our cohort of mildly hypertensive subjects.

In conclusion, mildly hypertensive patients did not present with increased plasma vWF levels or vWF function measured by collagen binding capacity. This questions the suitability of vWF levels as a marker for endothelial dysfunction at an early disease state and indicates that, at the early onset of hypertension, vWF is not a target of LC n-3 PUFAs to mediate their cardioprotective effects at the concentration and duration used in this study.

## Figures and Tables

**Table 1:** Subject demographics and clinical data for the different treatment groups.

Measurements	Treatment groups			
	Hypertensive n-3 PUFAs	Hypertensive placebo	Normotensive n-3 PUFAs	Normotensive placebo
Gender (F/M)	1/5	3/6	6/7	9/5
Age (years; mean ± SD)	45.7 ± 11.27	57.8 ± 10.29	51.1 ± 8.84	48.8 ± 8.84
BMI (kg/m^2^; mean ± SD)	33.5 ± 3.64^*^	28.6 ± 5.01	25.9 ± 4.04	27.0 ± 3.97
Medications				
None	*n* = 6	*n* = 4	*n* = 10	*n* = 9
NSAIDs		*n* = 1^∧^		*n* = 1
Antidepressant		*n* = 3	*n* = 1	*n* = 2
HRT		*n* = 1^∧^	*n* = 1	
Contraceptive pill		*n* = 1		
Antiallergy			*n* = 1	*n* = 1
Paracetamol		*n* = 1^#^		
Thyroxine				*n* = 1
Glucosamine		*n* = 1^#^		
Pygeum		*n* = 1^#^		
Methotrexate		*n* = 1^#^		
Smoking status				
Current	*n* = 2	*n* = 2	*n* = 2	*n* = 1
Never	*n* = 3	*n* = 4	*n* = 8	*n* = 5
Previous	*n* = 1	*n* = 3	*n* = 3	*n* = 8
Blood pressure (mmHg)				
SBP, baseline	142.92 ± 3.14^**^	142.69 ± 2.35^**^	115.20 ± 2.07	116.60 ± 2.50
SBP, week 12	138.43 ± 3.63^**^	139.94 ± 3.58^**^	112.38 ± 2.59	115.27 ± 2.76
DBP, baseline	91.44 ± 3.74^**^	85.75 ± 3.47^**^	74.07 ± 1.29	73.88 ± 1.59
DBP, week 12	90.68 ± 3.28^**^	85.68 ± 3.03^**^	73.01 ± 1.72	73.21 ± 2.15
Serum lipids (mM)				
TC, baseline	4.35 ± 0.32	4.78 ± 0.43	4.93 ± 0.31	4.96 ± 0.33
TC, week 12	4.49 ± 0.34	4.69 ± 0.45	5.21 ± 0.33	5.04 ± 0.29
TG, baseline	1.33 ± 0.18	1.26 ± 0.34	1.06 ± 0.17	0.88 ± 0.10
TG, week 12	1.05 ± 0.13	0.95 ± 0.24	0.90 ± 0.14	0.99 ± 0.11
HDL-C, baseline	1.11 ± 0.13	1.48 ± 0.26	1.54 ± 0.17	1.67 ± 0.14
HDL-C, week 12	1.26 ± 0.12	1.66 ± 0.28	1.55 ± 0.18	1.70 ± 0.15
LDL-C, baseline	0.87 ± 0.20	0.93 ± 0.17	1.06 ± 0.13	1.10 ± 0.15
LDL-C, week 12	0.99 ± 0.15	0.95 ± 0.12	1.25 ± 0.13	1.07 ± 0.12

n-3 PUFAs: omega-3 polyunsaturated fatty acids; BMI: body mass index; NSAIDs: nonsteroidal anti-inflammatory drugs; HRT: hormone replacement therapy; ^∧,#^multiple medications taken by the same subject; BP: blood pressure; SBP: systolic blood pressure; DBP: diastolic blood pressure; SL: serum lipids; TC: serum total cholesterol; TG: serum triglycerides; HDL-C: serum high density lipoprotein cholesterol; LDL-C: serum low density lipoprotein cholesterol; ^*^ *P* < 0.05 (hypertensive n-3 PUFA versus normotensive n-3 PUFA treatment group; one-way ANOVA); ^**^ *P* < 0.001 (hypertensive versus normotensive at baseline and week 12; one-way ANOVA). Data are expressed as mean ± SEM unless indicated otherwise.
